# The complete mitochondrial genome of the peppermint shrimp *Lysmata wurdemanni* (Decapoda: Lysmatidae)

**DOI:** 10.1080/23802359.2021.1937364

**Published:** 2021-06-15

**Authors:** Maisha T. Epps, Rosemary T. Nguyen, Kristina L. Samborski, Viktoria E. Bogantes, Alexis M. Janosik

**Affiliations:** Biology Department, University of West Florida, Pensacola, FL, USA

**Keywords:** mtDNA, Gulf of Mexico, Decapod, Hermaphroditic, *Lysmata wurdemanni*

## Abstract

In this study, the complete 16,979 bp mitochondrial genome of *Lysmata wurdemanni* (Gibbes, 1850) was determined from a specimen collected from Apalachee Bay, U.S. The mitogenome contains 37 genes, and consists of 32.33% A, 35.01% T, 19.55% C, and 13.10% G, with a total G + C content of 32.65%. A maximum likelihood phylogenetic tree based on mitochondrial protein-coding genes suggested *L. wurdemanni* is clustered with *Lysmata vittata* and *Lysmata amboinensis*, based on available mitochondrial sequences of relatives. These data are useful in determining phylogenetic relationships between Lysmatidae and Thoridae. The sequenced mitochondrial genome may also assist in understanding evolutionary distinctions and breeding strategies in hermaphroditic species.

*Lysmata wurdemanni* (Gibbes 1850) also known as ‘peppermint shrimp’ is a species of cleaner shrimp native to North American Atlantic Ocean waters (Rhyne and Lin 2006). Cleaner shrimp are ecologically important to coral reef habitats in part because of the symbiotic relationship they form with reef fish (Zhang et al. 1998; Lin and Zhang 2001). Specifically, they consume parasites from reef fish helping to support longevity and health of the reef (Lin and Zhang 2001). Additionally, *L. wurdemanni* has economic value as a popular species in the aquatic trade industry because of its decorative appearance and easy aquarium maintenance (Zhang et al. 1998). Though *L. wurdemanni* is frequently traded in the aquarium industry, other individuals are often misidentified under this species’ name (Baeza and Behringer 2017) due to difficulty in cleaner shrimp identification from closely resembling species. Studies have redefined the *Lysmata* genus, naming four new species and relabeling two known species based on morphological characteristics (Rhyne and Lin 2006). This paper includes the first full mitochondrial DNA sequence of *L. wurdemanni*. The mitochondrial genome of *L. wurdemanni* can serve as an additional tool to help confirm species identification and support taxonomic resolution of *L. wurdemanni* and related species.

*Lysmata wurdemanni* was collected in the intertidal zone of Apalachee Bay, Wakulla county, Florida, United States (30°04′03.7″N, 84°08′22.5″W) and preserved in 95% ethanol. The specimen is deposited in the invertebrate collection at Florida Museum of Natural History (www.floridamuseum.ufl.edu, John D. Slapcinsky, slapcin@flmnh.ufl.edu) under voucher number Arthropoda 058273. Genomic DNA was extracted using the DNeasy Blood and Tissue kit (Qiagen). DNA libraries were constructed using Illumina HiSeq (Illumina, San Diego, CA), and were sequenced using HiSeq platform, with 250-bp paired-end reads at the Hubbard Center for Genomics, Sequencing Core Facility (Durham, NH). Sequencing resulted in 6,790,832 raw reads, which were trimmed and normalized using Geneious Prime V. 2021.0.3. *De novo* assembly was conducted with Geneious Prime V. 2021.0.3 (https://www.geneious.com). The assembled genome was annotated with MITOS2 (Bernt et al. 2013).

The completed mitochondrial genome of *L. wurdemanni* was 16,979 bp in length (GenBank accession number: MZ144584) and contained 13 protein-coding genes (PCGs), 22 tRNA, and 2 rRNA (16S and 12S). Of the 37 total genes, 23 were coded by the heavy strand and 14 were coded by the light strand. For the 14 PCGs, the most common shared start codon was ATG (cox2, atp6, cox3, nad4, nad4l, cob), followed by ATA (atp8, nad6, cox1, nad1) and ATT (nad3, nad5, nad2). The most common termination codon was TAA (cox2, atp8, atp6, cox3, nad5, nad4, nad4l, nad6, cob, nad1, nad2, cox1). Overall mitochondrial base composition was 32.33% A, 35.01% T, 19.55% C, and 13.10% G, with a total G + C content of 32.65%, similar to its closest relative *L. amboinensis*, which has a G + C content of 35.96%. A more distantly related species *Thor amboinensis* has a G + C content of 26.90%, which is lower than both *L. wurdemanni* and *L. amboinensis.*

To determine the phylogenetic relationship of *L. wurdemanni*, a concatenated dataset of 13 PCGs from closely related species available from GenBank was used for tree building. The dataset includes two other species of Lysmatidae and two species of Thoridae, *Austinogebia edulis* (Upogebiidae) was used as the outgroup. A maximum-likelihood phylogenetic tree was constructed using MEGA X (Kumar et al. 2018) with 1000 bootstrap replicates (Figure 1). Phylogenetic analysis recovered two distinct groups, supporting the monophyly of Lysmatidae as previously suggested by De Grave et al (2014).

**Figure 1.**
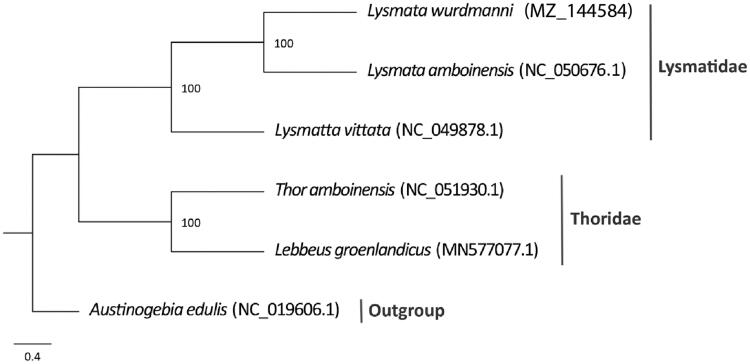
Phylogenetic tree of *L*ysmata *wurdemanni* and related species based on the maximum likelihood method.

## Data Availability

The data that support the findings are openly available in NCBI at (https://www.ncbi.nlm.nih.gov/), reference number (MZ144584). The associated BioProject, SRA, and Bio-Sample numbers are PRJNA731158, SRR14596716, and SAMN19272658 respectively.
